# Functional interaction between Ghrelin and GLP-1 regulates feeding through the vagal afferent system

**DOI:** 10.1038/s41598-020-75621-5

**Published:** 2020-10-28

**Authors:** Weidong Zhang, T. M. Zaved Waise, Koji Toshinai, Wakaba Tsuchimochi, Farhana Naznin, Md Nurul Islam, Ryota Tanida, Hideyuki Sakoda, Masamitsu Nakazato

**Affiliations:** 1 Division of Neurology, Respirology, Endocrinology, and Metabolism, Department of Internal Medicine, Faculty of Medicine, University of Miyazaki, 5200 Kihara, Kiyotake, Miyazaki 889-1692 Japan; 2 Toronto General Hospital Research Institute, UHN, Toronto, ON M5G 1L7 Canada; 3 Department of Sports and Fitness, Faculty of Wellness, Shigakkan University, 55 Nakoyama, Yokone, Obu 474-8651 Japan; 4 Department of Pharmacology and Therapeutics, University of Manitoba, Winnipeg, MB R3E 0J9 Canada; 5 Department of Endocrinology and Metabolism, Kanazawa University Graduate School of Medical Sciences, 13-1 Takara-machi, Kanazawa, Ishikawa 920-8640 Japan; 6 AMED-CREST, Japan Agency for Medical Research and Development, 1-7-1 Otemachi, Chiyoda-ku, Tokyo, 100-0004 Japan

**Keywords:** Neuroscience, Physiology, Endocrinology, Gastroenterology, Neurology

## Abstract

The gastrointestinal tract transmits feeding-regulatory signals to the brain via neuronal and hormonal pathways. Here we studied the interaction between the orexigenic gastric peptide, ghrelin, and the anorectic intestinal peptide, glucagon-like peptide 1 (GLP-1), in terms of feeding regulation via the vagal afferents. GLP-1 preadministration 30 min before ghrelin administration to rats and mice abolished ghrelin-induced food intake, while ghrelin preadministration abolished the anorectic effect of GLP-1. Ghrelin preadministration suppressed GLP-1-induced Fos expression in the nodose ganglia (NG). Electrophysiological assessment confirmed that the initially administered peptide abolished the vagal afferent electrical alteration induced by the subsequently administered peptide. Both the growth hormone secretagogue receptor (GHSR) and the GLP-1 receptor (GLP-1R) are co-localised in a major proportion of NG neurons that innervate the stomach. In these *Ghsr*^+^*Glp1r*^+^ neurons, ghrelin preadministration abolished the GLP-1-induced calcium response. Ghrelin generated a hyperpolarising current and GLP-1 generated a depolarising current in isolated NG neurons in a patch-clamp experiment. Ghrelin and GLP-1 potently influenced each other in terms of vagally mediated feeding regulation. This peptidergic interaction allows for fine control of the electrophysiological properties of NG neurons.

## Introduction

Gastrointestinal signals such as peptides and neural signals modulate feeding and energy homeostasis^1–4^. The vagus nerve, one of the cranial nerves, is composed of sensory afferents and motor efferents. The primary sensory neurons of the vagal afferents are located in the bilateral nodose ganglia (NG) that relay peripheral nerve signals from the gastrointestinal tract to the nucleus tractus solitarius (NTS) in the medulla oblongata. The vagal afferents are implicated in the regulation of acute feeding behavior and long-term energy balance^5,6^. The vagal afferents are anatomically heterogeneous, and their peripheral axons in the gastrointestinal tract form characteristic sensory endings that are specialized for detection of chemical (mucosal endings) or mechanical (primarily intraganglionic laminar endings (IGLEs) and intramuscular arrays) stimuli^7,8^. The latter two serve as mechanoreceptors to detect gastrointestinal distension and luminal stroking^7^. Enteroendocrine cells distributed throughout the gastrointestinal tract produce a variety of peptides, including glucagon-like peptide 1 (GLP-1), ghrelin, cholecystokinin, and peptide YY. These peptides regulate energy homeostasis by acting directly on target tissues as hormones or by activating intrinsic and extrinsic neurons in a paracrine manner^9,10^. Gastrointestinal peptides bind to their receptors which are synthesized in the cell body of the NG neurons located in the neck and are transported to vagal afferent terminals in the gastrointestinal tract^11,12^.

At the beginning of food intake, the activity of orexigenic signals is elevated while that of anorectic signals is suppressed. With ongoing food intake, anorectic signals overcome the orexigenic tone to terminate feeding. The anorectic peptide GLP-1, which is primarily released from enteroendocrine L cells in response to meals, functions as a satiety signal^13^. Plasma GLP-1 in humans reaches its peak level within 30 min after a meal^13^. GLP-1 delays gastric emptying in human and rodents^13,14^. Ghrelin is an orexigenic peptide that is secreted from gastric endocrine cells^15–17^. Plasma ghrelin levels in humans rise 1–2 h before the onset of a meal and fall to trough levels 30 min after the end of the meal^18,19^. Ghrelin increases gastric motility to enhance gastric emptying^20^.

GLP-1 receptor (GLP-1R) agonists have been introduced for the treatment of type 2 diabetes mellitus^3^. Bai et al. performed single-cell RNA sequencing of the NG neurons, morphological characterization, and behavioural analysis in mice to generate a map of the NG neurons that innervate the stomach and intestine^7^. GLP-1R-expressing neurons innervating the stomach react to gastric distension and transmit signals to the hypothalamus to suppress food intake^7,21^. The growth hormone secretagogue receptor (GHSR), a cognate ghrelin receptor expressed in the NG neurons, is transported to the stomach via the vagal afferents and stimulated by ghrelin^22–24^. Two different routes have been proposed to convey the gastric-derived ghrelin signals to the brain: the vagal afferent nerve and blood circulation. Ghrelin transmits hunger signals to the NTS via the vagal afferents^9,25,26^, however some studies denied this transmission. A study using subdiaphragmatic vagal deafferentation in rats demonstrated that vagal afferents are not necessary for the orexigenic effect of intraperitoneal ghrelin^27^. Another researcher group reported that GHSR restoration in the hindbrain and NG in *Ghsr*^−/−^ mice did not restore ghrelin-induced feeding^28^.

It has been proven that single NG neurons express multiple receptors for anorectic substances, for example GLP-1R, cholecystokinin A receptor, and leptin receptor^7,29^, but it remains unclear whether orexigenic GHSR coexists with anorectic receptors. Several lines of study have verified individual roles of GLP-1 and ghrelin in feeding regulation^14,17,30–34^; however, it remains largely unknown how these two counteracting peptides affect the vagal sensory system. Here, we investigated the effect of interaction between ghrelin and GLP-1 on feeding regulation governed by the vagal afferents. We showed that GLP-1R and GHSR were co-expressed in a major subpopulation of NG neurons that innervate the stomach in mice. We also studied the effects of the two peptides on intracellular calcium mobilization and the membrane potentials of isolated vagal afferent neurons.

## Results

### Preadministration of either ghrelin or GLP-1 abolishes feeding effect of GLP-1 or ghrelin

**Figure 1:**
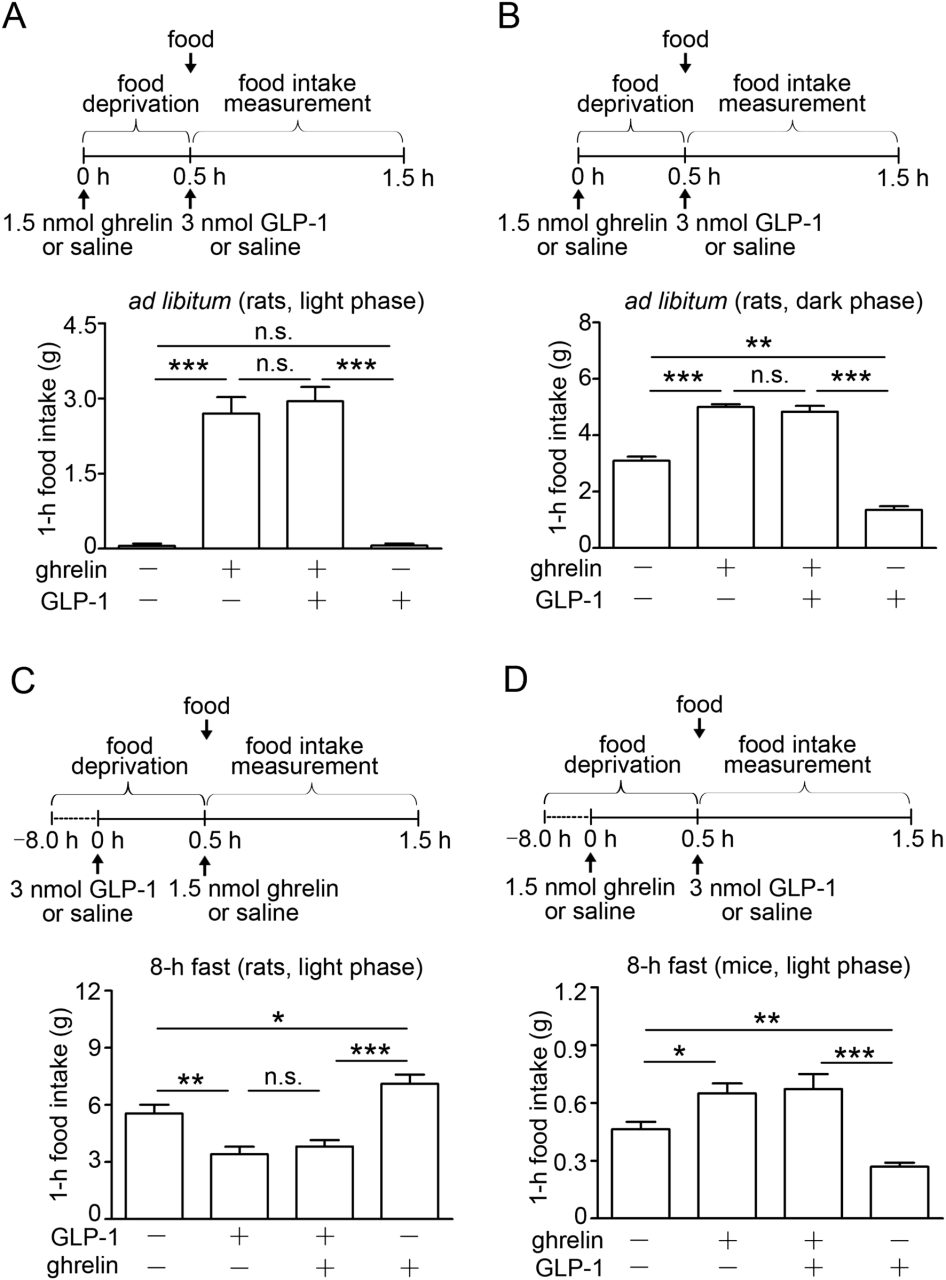

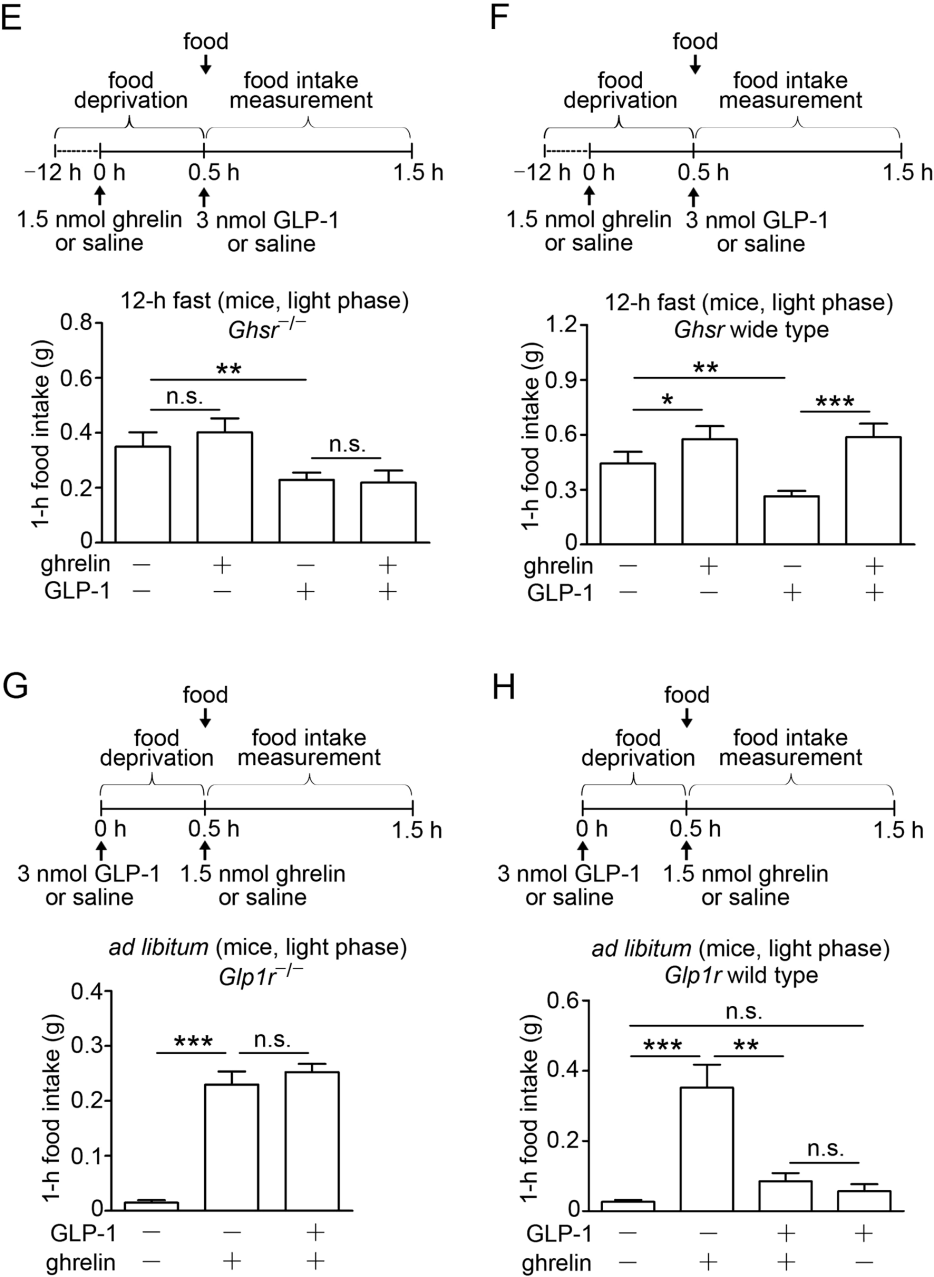
Interaction between ghrelin and GLP-1 on food intake. (**A**) Preadministration of ghrelin 30 min before GLP-1 abolished GLP-1‒induced suppression of feeding in rats fed ad libitum (n = 8). (**B**) GLP-1 administration to rats in the dark phase suppressed feeding, while preadministration of ghrelin 30 min before GLP-1 abolished GLP-1-induced suppression of feeding in rats fed ad libitum (n = 6). (**C**) Preadministration of GLP-1 30 min before ghrelin abolished ghrelin-induced feeding in rats fasted 8 h (n = 8). (**D**) Preadministration of ghrelin 30 min before GLP-1 abolished GLP-1‒induced suppression of feeding in mice fasted 8 h (n = 6). (**E**) Ghrelin did not induce food intake in *Ghsr*^−/−^ mice, and preadministration of ghrelin 30 min before GLP-1 failed to abolish GLP-1′s anorectic effect (n = 10). (**F**) Preadministration of ghrelin abolished GLP-1‒induced suppression of feeding in their wild type mice fasted 12 h (n = 10). (**G**) Preadministration of GLP-1 30 min before ghrelin to *Glp1r*^−/−^ mice fed ad libitum failed to abolish ghrelin-induced feeding (n = 8). (**H**) Preadministration of GLP-1 inhibited ghrelin-induced feeding in their wild type mice fed ad libitum (n = 8). All values are means ± SEM. ***P < 0.001, **P < 0.01, *P < 0.05, *ns* not significant.

To test whether the interaction between ghrelin and GLP-1 impacts on feeding regulation, we first designed feeding experiments in which these two peptides were administered in a 30-min interval. Food was given after the second peptide administration. In rats fed ad libitum, intravenous (i.v.) administration of ghrelin significantly enhanced 1-h food intake compared with saline injection (Fig. 1A). When food was given after GLP-1 administration, ghrelin kept its orexigenic effect (Fig. 1A). GLP-1 administration to rats in the dark phase suppressed feeding, while preadministration of ghrelin 30 min before GLP-1 also stimulated food intake (Fig. 1B). In 8-h fasted rats, GLP-1 significantly reduced food intake compared with saline injection, and ghrelin did not enhance feeding when it was administered 30 min after GLP-1 administration (Fig. 1C). We also studied food intake experiments in 8-h fasted mice. Ghrelin preadministration inhibited the anorectic effect of GLP-1 under this condition (Fig. 1D). In *Ghsr*^−/−^ mice fasted 12 h, ghrelin preadministration did not affect GLP-1‒induced anorexia (Fig. 1E), whereas ghrelin preadministration to their wild-type mice abolished GLP-1‒induced anorexia (Fig. 1F). When GLP-1 was administered to *Glp1r*^−/−^ mice fed ad libitum 30 min before ghrelin, GLP-1 did not affect ghrelin-induced feeding (Fig. 1G), whereas GLP-1 preadministration to their wild-type mice abolished ghrelin-induced feeding (Fig. 1H). To determine how long ghrelin and GLP-1 interact on feeding, we next administered the two peptides to mice in a 60-min interval. The results were different from those in studies in which they were administered in a 30-min interval. The peptide administered 60 min after the first peptide administration exhibited its proper effect in both cases with ghrelin and GLP-1 (Supplementary Fig. S1).

### The first peptide disappears from the plasma 30 min after its administration

We determined plasma profiles of ghrelin and GLP-1 administered to rats. When GLP-1 was administered first, its plasma concentration reached the peak at 5 min and returned to the base at 30 min when ghrelin was administered (Supplementary Fig. S2A). When ghrein was administered first, its plasma concentration also reached the peak at 5 min and returned to the base at 30 min when GLP-1 was administered (Supplementary Fig. S2B).

### GHSR and GLP-1R are expressed in NG neurons and transported within the vagal afferents

**Figure 2:**
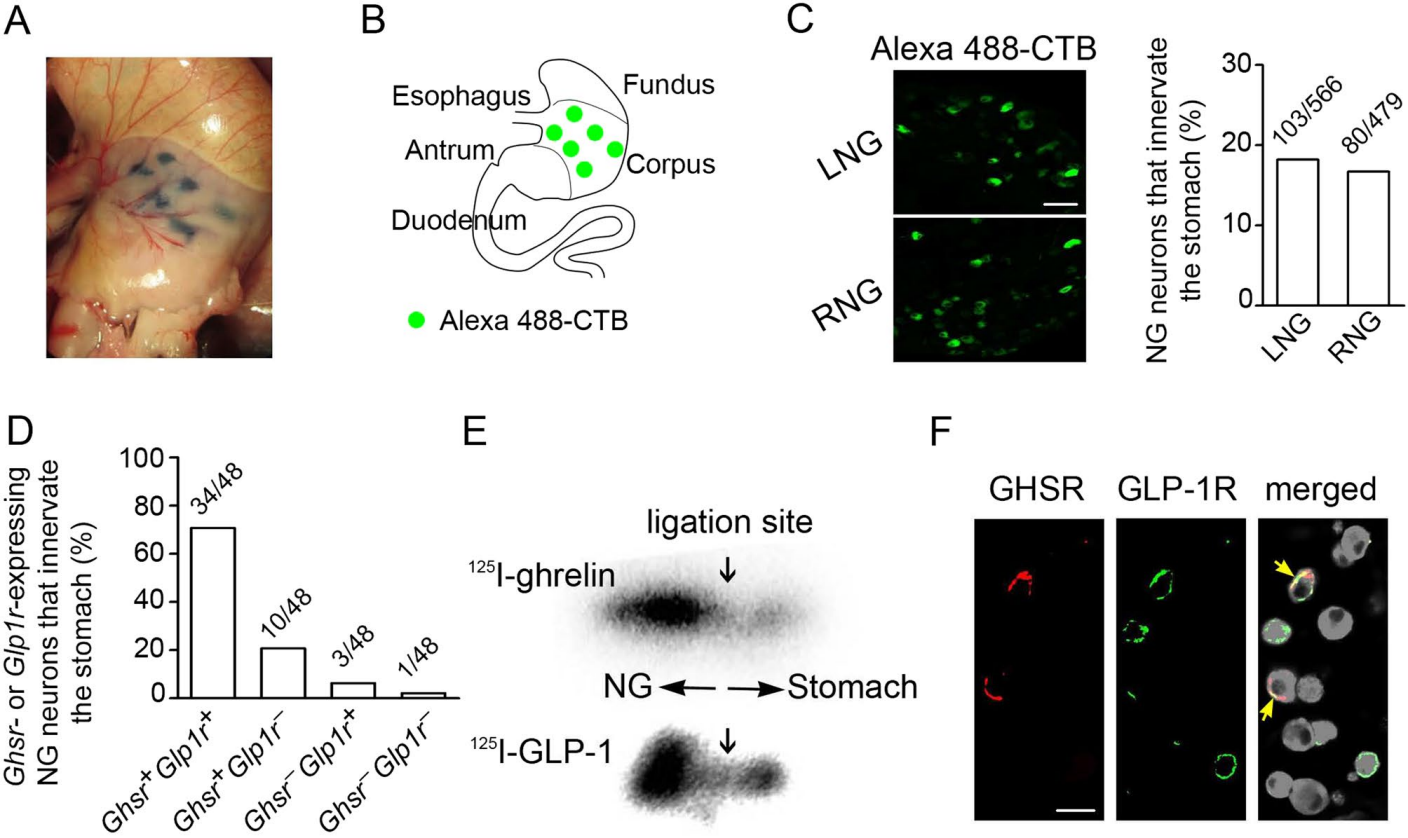
Transportation of GHSR and GLP-1R through the vagal afferent nerve in mice. (**A**) Representative photo to demonstrate multiple injection sites of Alexa Fluor 488-conjugated CTB in blue-black color at the gastric corpus. (**B**) Schematic illustration of injection sites of Alexa 488-CTB in green color at the ventral gastric corpus. (**C**) Representative images of left (LNG) and right (RNG) NG neurons labeled with Alexa Fluor 488-CTB transported from the gastric corpus. Percentage of stomach-projecting NG neurons (LNG; n = 566, RNG; n = 479). (**D**) Percentage of G*hsr-* or G*lp1r-*expressing NG neurons expressing G*hsr* or G*lp1r* that innervate the stomach (n = 48). (**E**) Representative autoradiographs showing the bindings of ^125^I-ghrelin and ^125^I-GLP-1 on the NG side of the ligated vagal afferents. (**F**) Representative images of immunocytochemistry of GHSR (red), GLP-1R (green), and their colocalization (arrows in yellow) in dispersed NG neurons. Scale bars 50 µm (**C**) and 25 µm (**F**).

To determine whether a distinct subset of NG neurons received signals from the gastric corpus, we performed a retrograde tracer study in mice. Right and left NG neurons were examined by fluorescence microscopy after injections of Alexa Fluor 488-conjugated cholera toxin B (CTB) into both ventral and dorsal sides of the gastric corpus (Fig. 2A, B). The number of NG neurons labeled with Alexa 488-CTB retrogradely transported from the stomach were nearly equal (18.2% of 566 left NG neurons and 16.7% of 479 right NG neurons) (Fig. 2C). To investigate the expression of G*hsr* and *Glp1r*, NG neurons labeled with Alexa 488-CTB were collected and studied by single-neuron mRNA analysis. Both *Ghsr-* and *Glp1r*-expressing neurons account for 70.8%, G*hsr* alone 20.8%, *Glp1r* alone 6.3%, neither of them 2.1% (Fig. 2D). To examine the transportation of GHSR and GLP-1R within the vagal afferents, we studied bindings of ^125^I-ghrelin and ^125^I-GLP-1 in the vagal segments. The transportation of these receptors from the NG neurons towards the afferent endings were blocked by ligation of the vagal segments. Radioactivities of both peptides were detected at the proximal to ligature (Fig. 2E). We applied immunocytochemistry to study colocalization of GHSR and GLP-1R in isolated NG neurons. Dispersed NG neurons of mice were double stained with anti-GLP-1R and anti-GHSR antibodies. Both receptors were observed in NG neurons in wild-type mice, whereas non-specific staining was not found in neither *Glp1r*^−/−^ or *Ghsr*^−/−^ mice (Fig. 2F and Supplementary Fig. S3). Subpopulations of NG neurons in wild-type mice expressed the both receptors (Fig. 2F).

### Preadministration of ghrelin or GLP-1 affects vagal nerve activity

**Figure 3:**
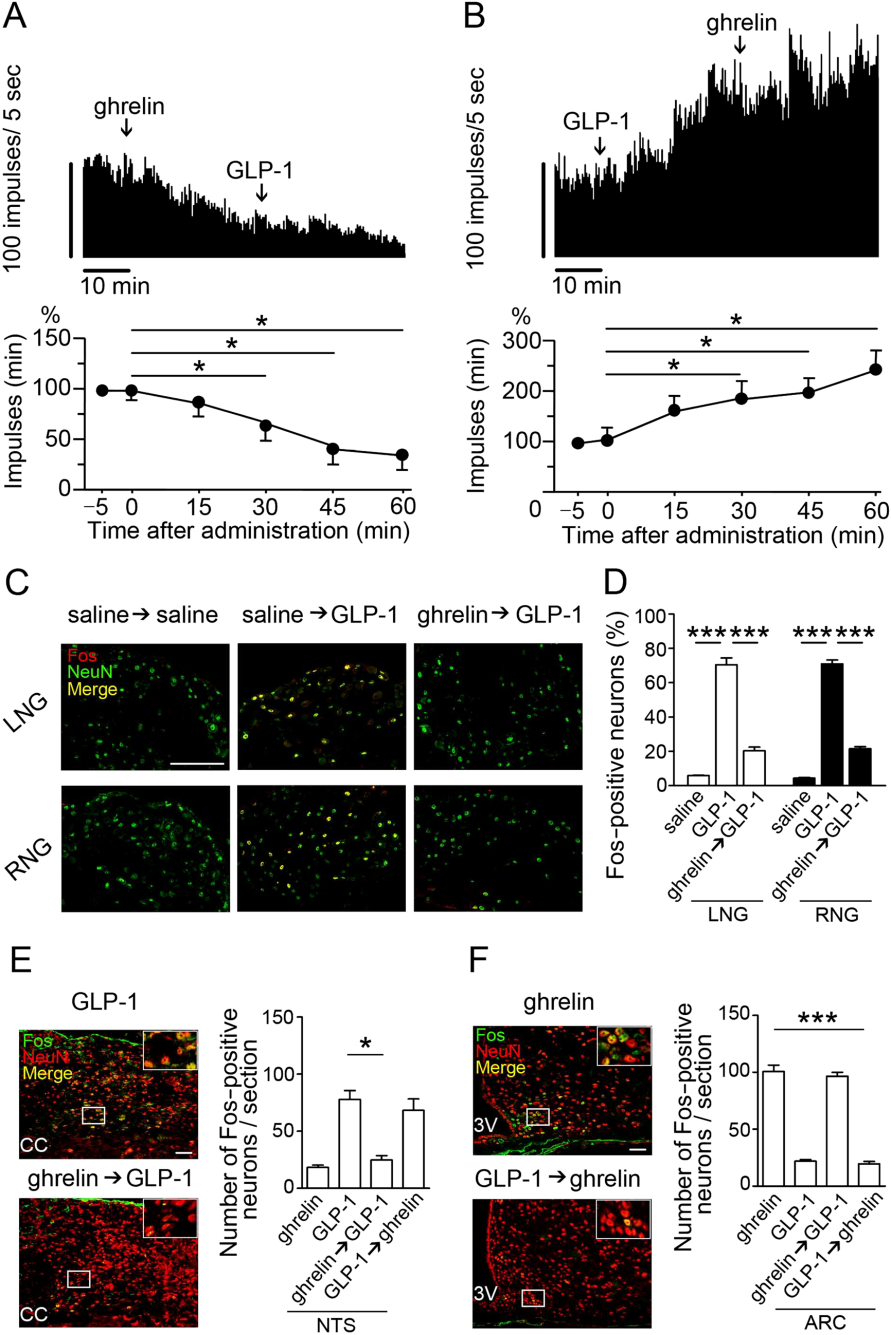
Interactive roles of ghrelin and GLP-1 on the vagal afferent nerve activity and Fos expression in NG, NTS, and hypothalamic ARC. (**A**) Representative records of gastric vagal afferent discharge rates in the upper panel. GLP-1 administration after ghrelin to rats did not activate gastric vagal afferent activity (n = 3). (**B**) Ghrelin administration after GLP-1 to rats did not attenuate gastric vagal afferent activity (n = 3). (**C**) Representative fluorescence images of GLP-1-induced Fos expression (red) in the NG neurons (green) in response to the saline, GLP-1 and GLP-1 after ghrelin in mice (n = 3). (**D**) Average percentage of Fos-positive neurons in LNG and RNG. GLP-1 induced Fos expression in the NG neurons. Ghrelin preadministration 30 min before GLP-1 significantly attenuated GLP-1-induced Fos expression in the NG neurons. (**E**,**F**) Representative fluorescence images of GLP-1- or ghrelin-induced Fos expression (green) in the NTS (**E**) and ARC (**F**) neurons (red). Ghrelin preadministration 30 min before GLP-1 significantly attenuated GLP-1-induced Fos expression in the NTS. GLP-1 preadministration 30 min before ghrelin significantly attenuated ghrelin-induced Fos expression in the ARC (n = 4). Magnified photographs represent Fos expression in the neurons (yellow in white box). CC, central canal; 3 V, third ventricle. Scale bars (**C**,**E**), 50 µm. All values are means ± SE. ***P < 0.001, *P < 0.05.

To investigate the interactive role of ghrelin and GLP-1 on the vagal afferent nerve activity, we assessed the multi-unit nerve discharge of the gastric vagal afferent of rats administered ghrelin and GLP-1. Ghrelin attenuated the vagal afferent activity (Fig. 3A), whereas GLP-1 enhanced it (Fig. 3B). When GLP-1 was administered 30 min after ghrelin, GLP-1-enhanced activity was not observed (Fig. 3A). Conversely, ghrelin administration 30 min after GLP-1 did not attenuate GLP-1-enhanced afferent activity (Fig. 3B).

We next studied Fos induction as a proxy for neuronal activation in mice. GLP-1 induced Fos in NG neurons (left 70.5% and right 71.0%) (Fig. 3C,D). This Fos expression was reduced by ghrelin administration before 30 min (left NG 21.7% and right NG 20.4%) (Fig. 3C,D). By contrast, ghrelin administration before 60 min did not affect GLP-1-induced Fos (left NG 68.5% and right NG 69.8%) (Supplementary Fig. S4). This finding supports the result in the feeding study operated in a 60-min interval in mice (Supplementary Fig. S1). GLP-1 also induced Fos in the NTS (Fig. 3E). Ghrelin preadministration abolished GLP-1-induced Fos in the NTS (Fig. 3E). Ghrelin induced Fos in the hypothalamic arcuate nucleus (ARC) (Fig. 3F). GLP-1 preadministration abolished ghrelin-induced Fos in the ARC (Fig. 3F).

### Preadministration of ghrelin or GLP-1 affects calcium response modulated by the second peptide administration and the two peptides have opposite effects on current–voltage

**Figure 4:**
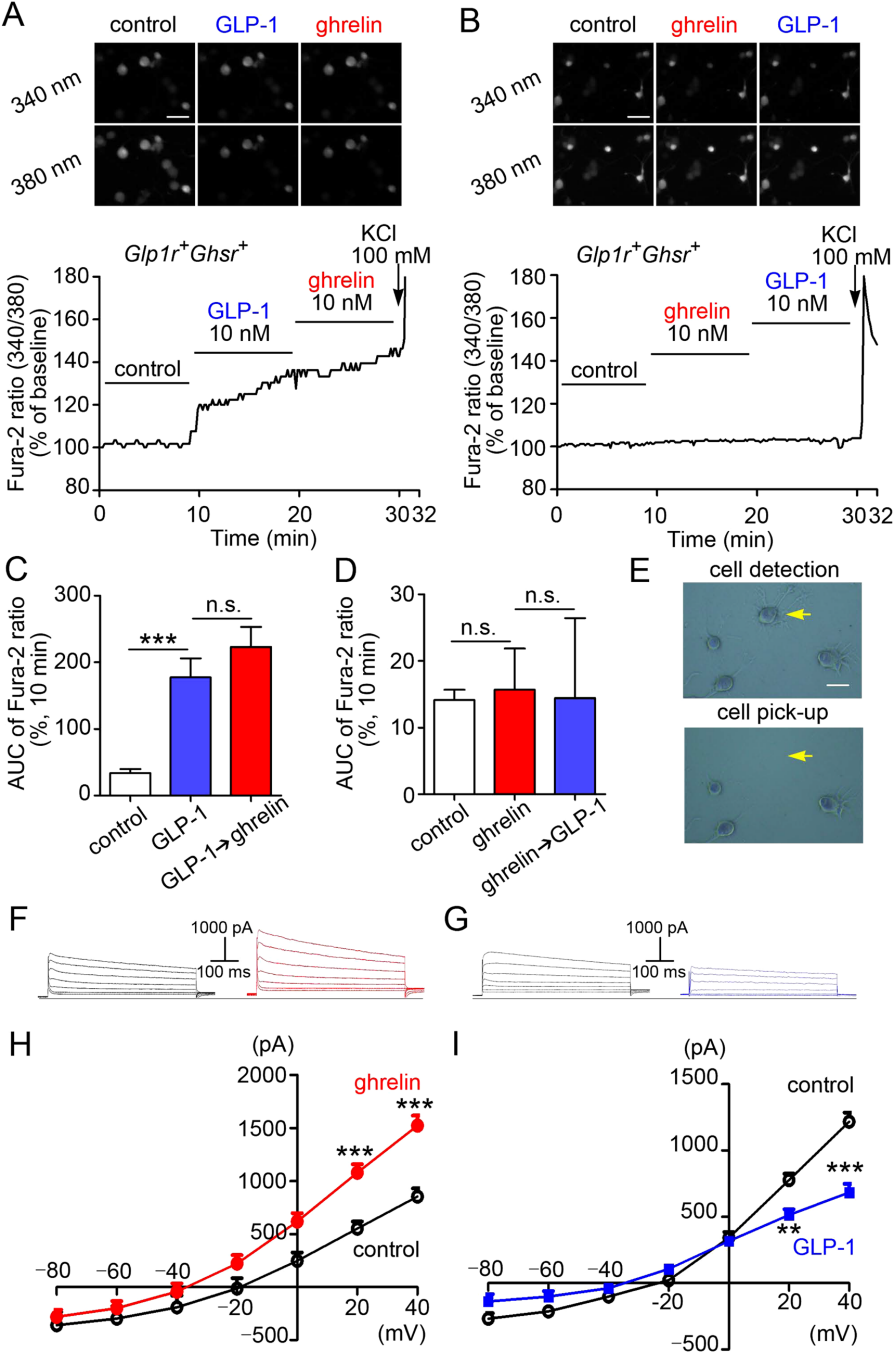
Effects of ghrelin and GLP-1 on calcium response and current–voltage in NG neurons. (**A**,**B**) Representative fluorescence images and percent changes from the basal 340/380 fluorescence ratio of calcium response to ghrelin- or GLP-1-induced activation in NG neurons. In *Glp1r*^+^*Ghsr*^+^ neurons, ghrelin administration after GLP-1 failed to suppress GLP-1-induced calcium responses (**A**, n = 10), and GLP-1 administration after ghrelin failed to increase calcium responses (**B**, n = 10). (**C**,**D**) Average areas under the curve (AUC) for [Ca^2+^]_i_ in **A** (**C**) and **B** (**D**). (**E)** GLP-1- or ghrelin-responsive neurons were collected after calcium imaging study to determine their *Glp1r* and *Ghsr* mRNA expression (yellow arrow). (**F**,**G**) Effects of ghrelin (**F**) and GLP-1 (**G**) on total outward currents of primary cultured NG neurons. (**H**,**I**) Changes of I/V relationships in response to ghrelin or GLP-1 administration. Ghrelin generated a hyperpolarising current and GLP-1 generated a depolarising current at both 20 and 40 mV (n = 10). Scale bars, 50 µm (**A**,**B**), and 25 µm (**E**). All values are means ± SE. ***P < 0.001, **P < 0.01, *ns* not significant.

To study neuronal activation of GLP-1 and ghrelin on the vagal afferents, we measured calcium response in single neurons isolated from mouse NG. GLP-1 evoked calcium response in *Glp1r* ^+^*Ghsr*^+^ neurons and ghrelin 10 min after GLP-1 did not affect the response (Fig. 4A,C). GLP-1 10 min after ghrelin failed to evoke calcium response in *Glp1r*^+^*Ghsr*^+^ neurons (Fig. 4B,D). GLP-1 evoked calcium response in *Glp1r*^+^*Ghsr*^−^ neurons (Supplementary Fig. S5A), but not in *Glp1r* ^−^*Ghsr*^−^ neurons (Supplementary Fig. S5B). Ghrelin did not evoke calcium response in *Glp1r*^−^*Ghsr*^+^ neurons (Supplementary Fig. S5E, F). After calcium measurement, all neurons were individually collected to study *Glp1r* and *Ghsr* expression by qRT-PCR (Fig. 4E).

We next performed whole-cell voltage-clamp study to examine the electrical properties of the NG neurons in response to ghrelin or GLP-1 administration. Current responses of the NG neurons to voltage clamp (− 80 to 40 mV) were used to generate the I/V relationships of ghrelin- or GLP-1-induced responses. Ghrelin generated hyperpolarizing current (Fig. 4F,H) and GLP-1 generated depolarizing current (Fig. 4G,I) in *Ghsr-* and *Glp1r-*expressing NG neurons, respectively.

## Discussion

This study demonstrated how vagal afferents modulate the functional interaction between GLP-1 and ghrelin in NG neurons to regulate feeding. The administration of ghrelin and GLP-1 at a 30-min interval abolished the feeding effect of the peptide administered second; however, when they were administered at a 60-min interval, this abrogation did not occur. This finding was consistent with the fact that ghrelin preadministration reduced GLP-1-induced Fos expression in the NTS when administered at a 30-min interval but not at a 60-min interval. Here, when the two peptides were administered at a 30-min interval, the plasma concentration of the first peptide returned to baseline upon administration of the second peptide. This suggests that the first peptide did not affect the second peptide as a circulating hormone.

Another possibility of the direct effects of GLP-1 and ghrelin in the brain has been proposed since both peptides cross the brain blood barrier^25,35^. Further studies are needed how peripherally administered ghrelin and GLP-1 activate the neurons in the brain; directly via their respective receptors, via the vagal afferents or both.

Luminal stroking and circular stretching of the stomach lead to feelings of hunger and satiety, respectively^3,7^. In humans, GLP-1 inhibits meal-induced gastrointestinal motility via vagal pathways^13^. GLP-1R-expressing NG neurons functions as mechanoreceptors that innervate gastric IGLEs and detect gastric distension^36^. Selective deletion of the GLP-1R in NG neurons accelerated gastric emptying in rats^14^. The GHSR is also expressed in tension-sensitive NG neurons that innervate the stomach^37^. Furthermore, ghrelin reduces the mechanosensitivity of gastric vagal afferents in mice and ferrets^38^. Together, ghrelin and GLP-1 may interact to regulate feeding by modulating gastric motility. The right and left vagal nerves enter the abdomen as two trunks that divide into distinct primary branches at the subdiaphragmatic esophageal level^39^. The ventral and dorsal surfaces of the stomach are innervated by gastric branches that originate from neurons in the right and left NG, respectively^39,40^. The small intestine is innervated by neurons from both the right and left NG^40^. In this study, a retrograde tracer experiment in which CTB was injected in equal into the ventral and dorsal sides of the stomach indicated that nearly the same number of neurons from the right (16.7%) and left (18.2%) NG innervated the stomach, supporting the branching pattern of the vagal gastric neurons. We also found four groups of stomach-projecting NG neurons on the basis of the expression of GHSR and GLP-1R (both GHSR- and GLP-1R-expressing neurons, 70.8%; GHSR alone, 20.8%; GLP-1 alone, 6.3%; neither, 2.1%). A major portion (91.6%) of stomach-projecting NG neurons expressed GHSR, supporting the previous finding of GHSR expression in 98% of tension-sensitive NG neurons that were retrogradely traced from the stomach^37^. To explore the effect of ghrelin and GLP-1 on the cellular excitability of *Glp1r*^+^*Ghsr*^+^ neurons, we studied these neurons’ intracellular signalling mechanisms.

Activation of GLP-1R, a G_αs_-coupled GPCR, increased cAMP concentrations in rat NG neurons and enhanced their excitability by suppressing potassium currents and enhancing membrane depolarization, thereby increasing intracellular calcium^41,42^. GHSR activates a G_αq/11_-coupled GPCR in the pituitary to stimulate intracellular calcium mobilization from the endoplasmic reticulum^33^, whereas in NG neurons, Grabauskas et al. showed that ghrelin-induced hyperpolarization was suppressed by pertussis toxin, a G_αi_ protein inhibitor^43^. They concluded that by activating the G_αi_-PI3K-Erk1/2-K_ATP_ pathway in NG neurons, ghrelin evoked potassium currents and thereby causes hyperpolarization^43^. GPCRs can bind to distinct classes of heterotrimeric G-proteins in different cell types. We here showed that ghrelin preadministration abolished the GLP-1-induced calcium response in *Glp1r*^+^*Ghsr*^+^ neurons, supporting that GHSR in NG neurons is coupled to G_αi_ protein. Using a patch-clamp experiment combined with single-neuron mRNA analysis, we verified that *Glp1r*^+^*Ghsr*^+^ neurons were depolarized by GLP-1 and hyperpolarized by ghrelin. These physiological effects on *Glp1r*^+^*Ghsr*^+^ neurons may modulate the anorectic or orexigenic signals that they convey to the brain.

In summary, we demonstrated that ghrelin and GLP-1 influence feeding regulation through their opposite effects on the cellular excitability of vagal afferent neurons. This peptidergic interaction sheds light on the fine control of feeding regulation.

## Methods

### Animals

Male C57BL/6 J mice (10- to 12-week-old; 22–28 g) and Wistar rats (9- to 10-week-old; 250–350 g) (Charles River Laboratories) were maintained in individual cages under controlled temperature (21–23 °C) and light (light on 08:00–20:00) conditions. Ghrelin receptor knockout mice (*Ghsr*^−/−^, kindly donated by Professor Smith RG, Department of Metabolism and Aging, Scripps Research Institute Florida^44^), GLP-1 receptor knockout mice (*Glp1r* ^−/−^; 10- to 12-week-old; 22–28 g; male) were kindly donated by Professor Yuichiro Yamada, Division of Endocrinology, Metabolism and Geriatric Medicine, Akita University Graduate School of Medicine, Japan. Mice and rats were maintained on a chow diet (CLEA Rodent Diet CE-2, CLEA Japan) with free access to food and water unless stated otherwise. When necessary, they were anesthetised with sodium pentobarbital (5 mg/kg mouse and 50 mg/kg rat body weight, i.p. administration, Abbot Laboratories). All animal experiments were approved by the Animal Care and Use Committee of University of Miyazaki and complied with the guidelines for the care and use of laboratory animals at University of Miyazaki.

### Feeding experiments

First, rats fasted for 8 h (02:00–10:00) received an i.v. injection of human GLP-1 (3 nmol/100 µl saline, CS Bio) or saline at 10:00, then they were administered human ghrelin (1.5 nmol/100 µl saline, Peptide Institute) or saline injection at 10:30. Food intake was determined from 10:30 to 11:30. Secondly, rats fed ad libitum received an i.v. ghrelin (1.5 nmol/100 µl) or saline injection at 10:00 or 19:30, then they were administered GLP-1 (3 nmol/100 µl) or saline injection at 10:30 or 20:00. Food intake was measured from 10:30 to 11:30 or 20:00 to 21:00. Thirdly, mice fasted for 8 h (02:00–10:00) received an i.p. injection of ghrelin (1.5 nmol/100 µl saline) or saline at 10:00, then they were administered human GLP-1 (3 nmol/100 µl saline) or saline at 10:30 and 1 h food intake (10:30–11:30) was measured. Fourthly, *Ghsr*^−/−^ and their wild-type mice fasted for 12 h (22:00–10:00) were administered ghrelin (1.5 nmol/50 µl saline, i.p.) at 10:00, then they were administered GLP-1 (3 nmol/50 µl saline, i.p.) or saline injection at 10:30, and food intake was measured from 10:30 to 11:30. Finally, *Glp1r* ^−/−^ and their wild-type mice were administered GLP-1 (3 nmol/50 µl, i.p.) or saline at 10:00, subsequently given a ghrelin (1.5 nmol/50 µl, i.p.) or saline injection at 10:30, food intake was measured from 10:30 to 11:30. These feeding experiments were performed using a crossover design with 2–3 days apart between experimental days.

### Jugular vein cannulation

Core body temperature of anesthetized rats was maintained at 37 °C using a heating blanket. A sterilised i.v. catheter (Braintree Scientific) containing pyrogen-free heparin saline (500 U/ml) was inserted into the jugular vein. Rats were acclimated to the experimental conditions by daily handling during 7 days. Only rats exhibiting progressive weight gain after surgery were used in the experiments.

### Pharmacokinetics analysis

Rats were given an i.v. injection of either GLP-1 (3 nmol/100 µl saline) or ghrelin (1.5 nmol/100 µl saline) into the right femoral vein, then 30 min later they were given the other peptide into the left femoral vein. Blood was withdrawn from the right jugular vein at:  1, 2.5, 5, 10, 20, 30, 32.5, 35, 40, 50, and 60 min after the first injection. Blood was collected into tubes containing 1.25 mg EDTA and aprotinin (500 kallikrein-inhibiting units/ml blood) (Wako Pure Chemical Industries), and all samples were centrifuged within 2 h of collection. For GLP-1 assay, DPP4 inhibitor (10 µl/ml) (Millipore) was added to blood immediately after collection. For ghrelin assay, 1 M HCl (100 µl/ml) was added to the plasma. All samples were stored at − 70 °C until analysis. GLP-1 was measured with a Glucagon-Like Peptide-1 (Active) ELISA kit (Millipore), and ghrelin in an automated enzyme immunoassay system (AIA-600 II Immunoassay Analyzer, TOSOH Bioscience).

### Culture preparation and immunocytochemistry

Bilateral NGs resected from mice were immersed in hibernateA media (BrainBits). Each ganglion was cut into five pieces, placed in HibernateA minus Ca^2+^ media (BrainBits) containing 1 mg/ml collagenase/dispase (Roche Diagnostics) and incubated for 90 min at 37 °C. Neurons were dispersed by gentle titration through pipettes and they were washed three times with fresh NbActive4 media (BrainBits) and plated onto poly-D-lysine/laminin (BD Bioscience)-coated coverslips, followed by incubation (5% CO_2_ in air at 37 °C) for 24 h. Cultures were rinsed in 10 mM phosphate buffered saline (PBS) and fixed with 4% paraformaldehyde (PFA) in phosphate buffer (PB). Ganglion neurons were incubated with the first primary antibodies; anti-GLP-1R or anti-GHSR at 4 °C overnight, and then with the second primary antibodies; anti-GHSR or anti-Pan Neuronal Marker (Supplementary Table S1) at 4 °C overnight. They were then reacted with corresponding Alexa Fluor 488- or 594-conjugated secondary IgG (Supplementary Table S1) at RT for 1 h. Neurons were observed under a fluorescence microscope (Olympus).

### Vagal branch ligation and autoradiography

Crushing ligation of the four branches (hepatic, celiac, ventral gastric, and dorsal gastric) of the ventral vagal trunk in C57BL/6 J mice was performed with suture thread. The vagal branch was excised 16 h later, embedded in Tissue-Tek O.C.T. compound, and frozen. Serial sections (6 µm) were cut using a cryostat along the longitudinal axis of the nerve and were mounted onto glass slides. After treating the sections at 37 °C with binding buffer (Composition in mmol L^−1^: HEPES 20, NaCl 150, MgCl_2_ 5, ethylene glycol-bis (β-aminoethyl ether)-*N*, *N*, *N*', *N*'-tetraacetic acid 1, and 0.1% BSA) for 60 min, we incubated the vagal branch with 15 nM ^125^I-ghrelin or 15 nM ^125^I-GLP-1 for 30 min. Nonspecific binding was determined in the presence of excess (15 µM) unlabelled ghrelin or GLP-1. Sections were exposed to an imaging plate (Fuji Film, Tokyo, Japan) for 24 h and analysed using an FLA-7000 phosphorimaging system (Fuji Film).

### Retrograde tracing studies

The abdomen of mice (n = 3) was exposed after a midline laparotomy. Alexa Fluor 488-conjugated CTB (CTB AF-48 0.5%, 1 µl; Molecular Probes) was injected beneath the serosal layers of the gastric corpus with a 5 µl Hamilton syringe (30-G needle). Six injections with equal spacing were made on ventral and dorsal sides of stomach. The needle was left in the places for 30 s to avoid dye leakage into the peritoneal cavity. After withdrawal of the needle, the area was swabbed to remove any excess tracer. Two days after, mice were anaesthetised and transcardially perfused with ice-cold PB followed by 4% PFA solution. Bilateral NGs were collected, embedded in Tissue-Tek O.C.T. compound (Sakura Finetek), and frozen on a block of dry ice. The NGs were cut longitudinally into 12-µm serial sections in a cryostat (Leica CM3050S) chilled at − 20 °C. Five sections from each mouse were mounted onto glass slides and observed under a fluorescence microscope (Olympus). To investigate percentage of NG neurons expressing G*hsr* or *Glp1r* that innervate the stomach, the dispersed NG neurons that indicated by Alexa 488-CTB were collected and used for mRNA analysis (see details in single-cell RT-PCR below).

### Fos immunohistochemical staining

Mice (n = 3) were administered i.p. injection of either GLP-1 (3 nmol/50 µl saline) or ghrelin (1.5 nmol/50 µl saline), then 30 min later they received the other peptide. The NG and brain were removed, fixed overnight in a fixative solution containing 4% PFA, cryoprotected in 0.1 M PB containing 20% sucrose and embedded in Tissue-Tek O.C.T. compound. The 10 µm sections were mounted on slides permeabilized with 0.3% triton X-100 in PBS (PBS-T). After permeabilization, antigens were retrieved by heating the sections in an autoclave for 1 h at 70 °C with a Histo VT one (Nakalai Teaque, Kyoto, Japan). All sections were blocked with 5% goat-serum in PBS-T. Three specimens each of NG, NTS and ARC were incubated for 48 h at 4 °C with c-Fos antiserum (Supplementary Table S1), then incubated for 1 h with Alexa Fluor 488- (NTS and ARC) or 594- (NG) conjugated anti-rabbit IgG at room temperature (Supplementary Table S1). To stain nuclei of the neurons, the specimens from NG were incubated with Alexa Fluor 488-conjugated Anti-NeuN IgG (Supplementary Table S1) at RT for 1 h, and the specimens from NTS and ARC were incubated overnight with Alexa Fluor 555-conjugated anti-NeuN IgG (Supplementary Table S1) at 4 °C. Images of immunofluorescence staining were observed using a Nikon C2 + confocal laser scanning microscope (Nikon, Tokyo, Japan). Fos-positive neurons in three specimens from each animal were counted using ImageJ.

### Electrophysiological recordings of the vagal nerve

Multi-unit neural discharge in gastric vagal afferent fibres was recorded extracellularly. Rats were anesthetised by an i.p. injection of urethane (1 g/kg) (Sigma-Aldrich). The electrophysiological study was performed entirely under anaesthetisation as described previously^45^. After the gastric branch of the vagal nerve was visualised, we placed filaments isolated from the peripheral cut end of the ventral branch for the recording of afferent nerve activity via a pair of silver wire electrodes connected through an alternating current-coupled differential amplifier (ER-1; Cygnus Technology) to PowerLab (AD Instruments). The nerve activities were recorded and analysed using LabChart v8 software (AD Instruments). First, ghrelin (1.5 nmol/200 μl saline) or GLP-1 (1 nmol/200 μl saline) was administered to rats through an i.v. catheter inserted into the inferior vena cava, and nerve discharges from the multiunit afferents were recorded for 30 min. Next, rats were given an i.v. injection of either GLP-1 (1 nmol/200 µl saline) or ghrelin (1.5 nmol/200 µl saline), and 30 min later the other peptide was administered. After the first administration, nerve discharges from the multi-unit afferents were analysed for 60 min.

### Measurement of calcium response

Ca^2+^ imaging was performed under continuous perfusion of NG neurons with Krebs–Ringer-Henseleit buffer (Composition in mmol L^−1^: NaCl 119, KCl 4.74, CaCl_2_ 2.54, MgCl_2_ 1.19, KH_2_PO_4_ 1.19, NaHCO_3_ 25, HEPES 10, pH 7.4) at a flow rate of 600 μl/min at 37 °C. NG neurons were loaded with 1 µM Fura-2 acetoxymethyl ester (Dojindo Labs) in NbActive4 media at 37 °C for 30 min. For Ca^2+^ measurements in an integrated fluorescence microscope (Keyence BZ-X700), images were captured at 10-s intervals; 340- and 380-nm excitation filters were used for Fura-2-AM dual-wavelength excitation ratio imaging^46^. NG neurons were stimulated with 10 nM either ghrelin or GLP-1, and 10 min later stimulated with the other peptide. At the end of experiments, cells were exposed to 100 mM KCl (Nacalai Tesque) for 2 min. Cells exhibiting more than 200% of the 340/380 fluorescence ratio in response to KCl were used for the analysis. All data were expressed as percent changes from the average of the 340/380 fluorescence ratio in response to ghrelin or GLP-1.

### Patch-clamp electrophysiological recordings

NG neurons were perfused in the bath solution (Composition in mmol L^−1^: NaCl 126.2, KCl 3.1, MgCl_2_ 2, CaCl_2_ 2, HEPES 10, glucose 10; pH 7.39, osmolarity 320 mOsm) with 10 nM ghrelin or 10 nM GLP-1. Whole-cell recordings were performed using patch pipettes of 3.5–5 MΩ resistance backfilled with a solution (Composition in mmol L^−1^: K gluconate 125, KCl 8, MgCl_2_ 4, HEPES 10, ATP 2, GTP 0.4, Na_2_-creatine phosphate 5; pH 7.23, osmolarity 300 mOsm). After obtaining a giga-seal, membrane access resistance was monitored for 5–10 min until it became stable at 10 MΩ or less. Data were acquired from isolated vagal sensory neurons of mice in Axopatch 1D amplifier (Molecular Devices) filtered at 1 kHz. Signals were digitised in analogue-to-digital converter (Axon Digidata 1322A; Molecular Devices), and analysed on a computer running pCLAMP 10 software (Molecular Devices).

### Single-cell RT-PCR

All neurons collected in retrograde tracing studies and used to assess calcium response and patch-clamp recording were picked up by the UnipicK + system (NeuroInDx) immediately after each measurement. RNA was isolated from single neurons with PicoPure RNA Isolation Kit (Applied Biosystems). qRT-PCR (primer sets, Supplementary Table S2) was conducted with SYBR Premix Ex Taq (2 ×) (Takara Bio Inc.) on a Thermal Cycler Dice Real-Time System II (Takara Bio Inc.). In the retrograde tracer studies, percentage of stomach-innervating NG neurons expressing *Ghsr* or *Glp1r* were analysed. In the patch-clamp recording, only *Ghsr*^+^ *Glp1r*^+^ neuron cells were used for the final analysis.

### Statistical analysis

Statistical analyses were performed by one-way ANOVA followed by Bonferroni’s post-test for multiple comparisons, as appropriate. When two mean values were compared, analyses were performed by unpaired *t*-test. All data are expressed as means ± s.e.m. A value of *P* < 0.05 was considered to be statistically significant.

## Supplementary information

Supplementary Information
