# Supplementary material for: Functional interaction between Ghrelin and GLP-1 regulates feeding through the vagal afferent system
Source: Sci Rep. 2020 Oct 28;10:18415. doi: 10.1038/s41598-020-75621-5 (PMC7595212; doi:10.1038/s41598-020-75621-5)
Supplement: Supplementary file 1 — Supplementary Information [file 41598_2020_75621_MOESM1_ESM.pptx]

## Slide 1
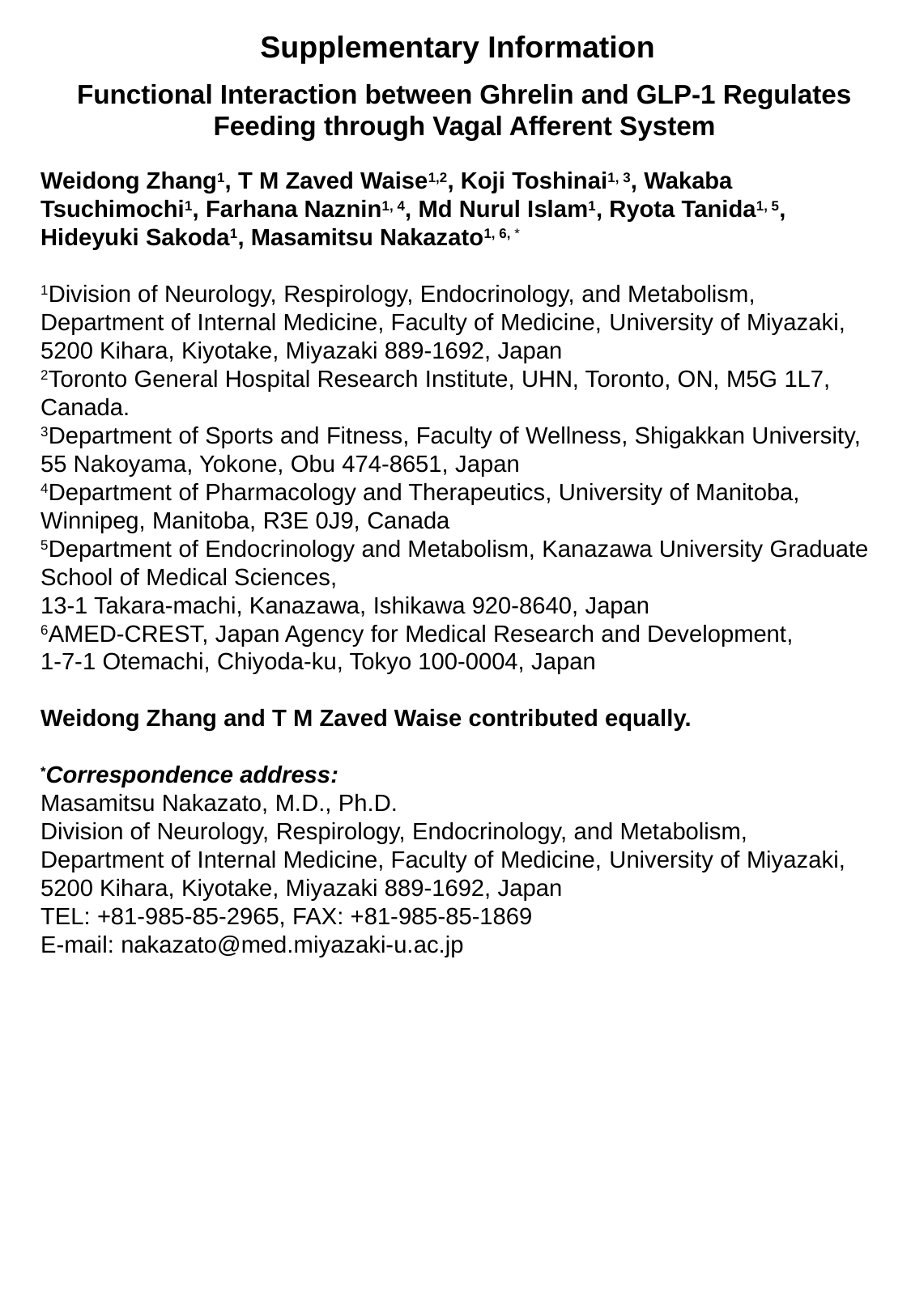

Supplementary Information
Functional Interaction between Ghrelin and GLP-1 Regulates Feeding through Vagal Afferent System
Weidong Zhang1, T M Zaved Waise1,2, Koji Toshinai1, 3, Wakaba Tsuchimochi1, Farhana Naznin1, 4, Md Nurul Islam1, Ryota Tanida1, 5, Hideyuki Sakoda1, Masamitsu Nakazato1, 6, *
1Division of Neurology, Respirology, Endocrinology, and Metabolism,
Department of Internal Medicine, Faculty of Medicine, University of Miyazaki,
5200 Kihara, Kiyotake, Miyazaki 889-1692, Japan
2Toronto General Hospital Research Institute, UHN, Toronto, ON, M5G 1L7, Canada.
3Department of Sports and Fitness, Faculty of Wellness, Shigakkan University,
55 Nakoyama, Yokone, Obu 474-8651, Japan
4Department of Pharmacology and Therapeutics, University of Manitoba, Winnipeg, Manitoba, R3E 0J9, Canada
5Department of Endocrinology and Metabolism, Kanazawa University Graduate School of Medical Sciences,
13-1 Takara-machi, Kanazawa, Ishikawa 920-8640, Japan
6AMED-CREST, Japan Agency for Medical Research and Development,
1-7-1 Otemachi, Chiyoda-ku, Tokyo 100-0004, Japan
Weidong Zhang and T M Zaved Waise contributed equally.
*Correspondence address:
Masamitsu Nakazato, M.D., Ph.D.
Division of Neurology, Respirology, Endocrinology, and Metabolism,
Department of Internal Medicine, Faculty of Medicine, University of Miyazaki,
5200 Kihara, Kiyotake, Miyazaki 889-1692, Japan
TEL: +81-985-85-2965, FAX: +81-985-85-1869
E-mail: nakazato@med.miyazaki-u.ac.jp

## Slide 2
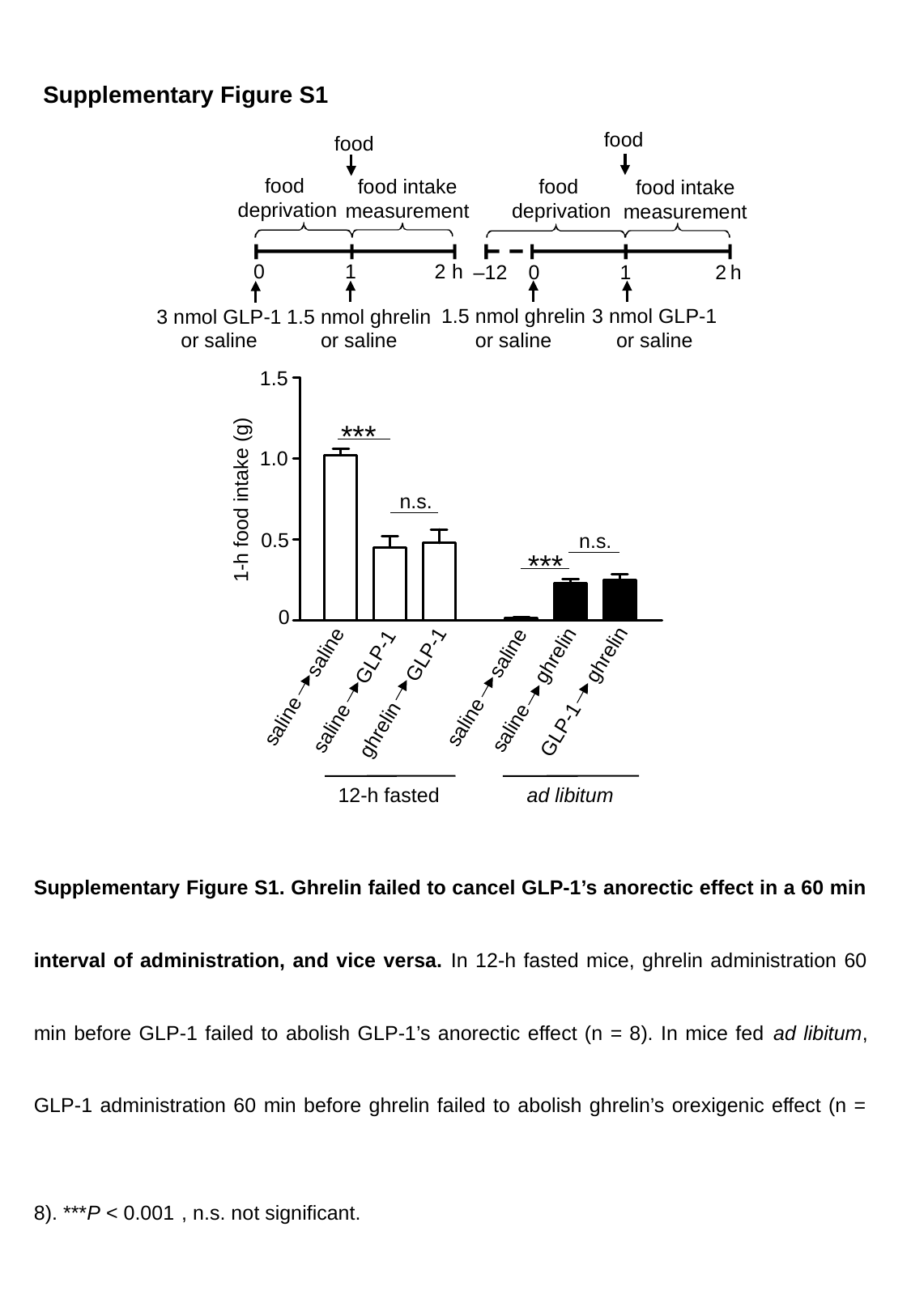

Supplementary Figure S1
food
food
food
deprivation
food
deprivation
food intake
measurement
food intake
measurement
 0
1
2 h
‒12
 0
1
2 h
1.5 nmol ghrelin
or saline
3 nmol GLP-1
or saline
1.5 nmol ghrelin
or saline
3 nmol GLP-1
or saline
1.5
***
1.0
1-h food intake (g)
n.s.
0.5
n.s.
***
0
saline saline
saline saline
saline ghrelin
saline GLP-1
GLP-1 ghrelin
ghrelin GLP-1
12-h fasted
ad libitum
Supplementary Figure S1. Ghrelin failed to cancel GLP-1’s anorectic effect in a 60 min interval of administration, and vice versa. In 12-h fasted mice, ghrelin administration 60 min before GLP-1 failed to abolish GLP-1’s anorectic effect (n = 8). In mice fed ad libitum, GLP-1 administration 60 min before ghrelin failed to abolish ghrelin’s orexigenic effect (n = 8). ***P < 0.001 , n.s. not significant.

## Slide 3
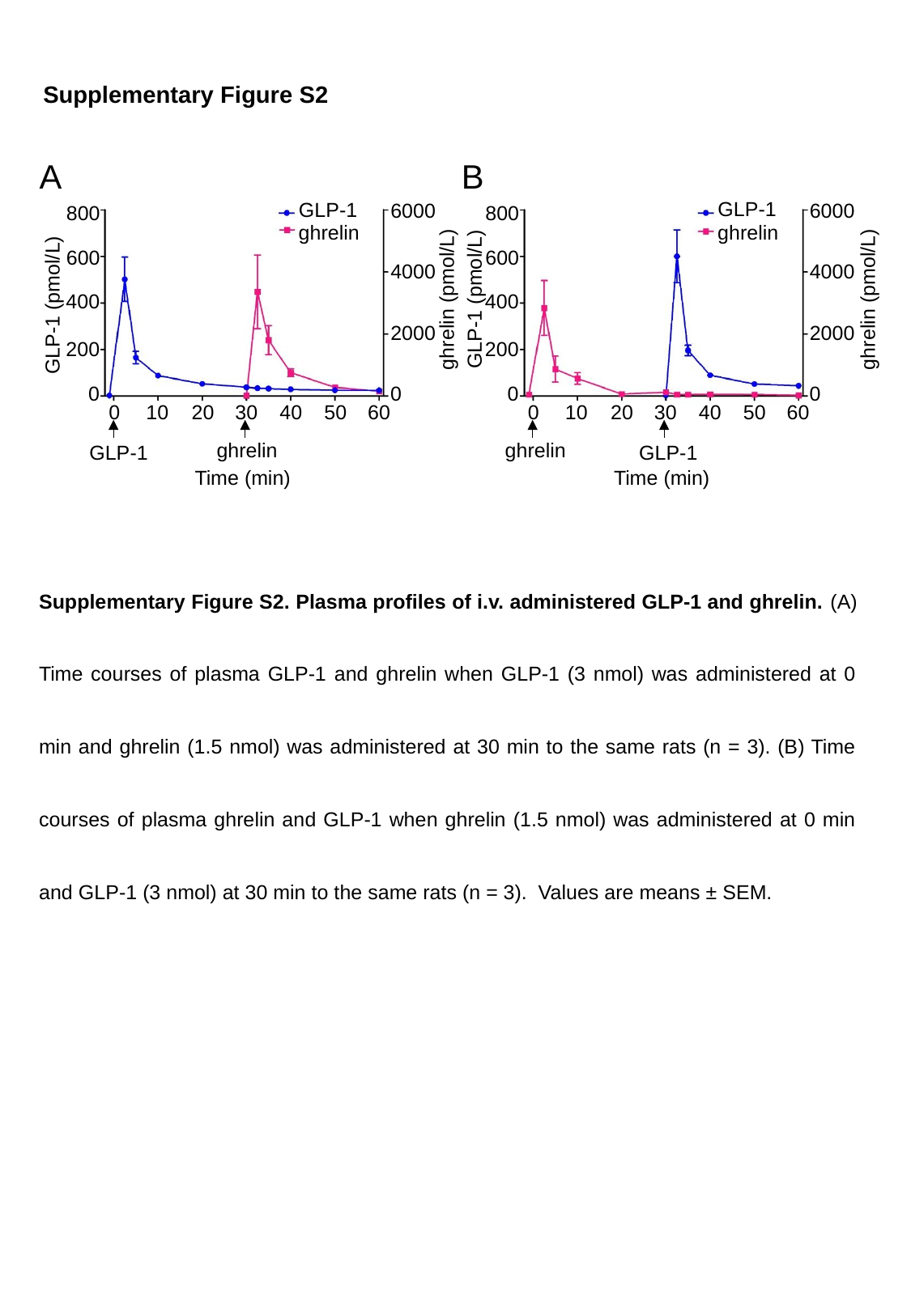

Supplementary Figure S2
A
B
GLP-1
GLP-1
6000
6000
800
800
ghrelin
ghrelin
600
600
4000
4000
GLP-1 (pmol/L)
ghrelin (pmol/L)
ghrelin (pmol/L)
400
400
GLP-1 (pmol/L)
2000
2000
200
200
0
0
0
0
0
0
10
20
30
40
50
60
10
20
30
40
50
60
ghrelin
ghrelin
GLP-1
GLP-1
Time (min)
Time (min)
Supplementary Figure S2. Plasma profiles of i.v. administered GLP-1 and ghrelin. (A) Time courses of plasma GLP-1 and ghrelin when GLP-1 (3 nmol) was administered at 0 min and ghrelin (1.5 nmol) was administered at 30 min to the same rats (n = 3). (B) Time courses of plasma ghrelin and GLP-1 when ghrelin (1.5 nmol) was administered at 0 min and GLP-1 (3 nmol) at 30 min to the same rats (n = 3). Values are means ± SEM.

## Slide 4
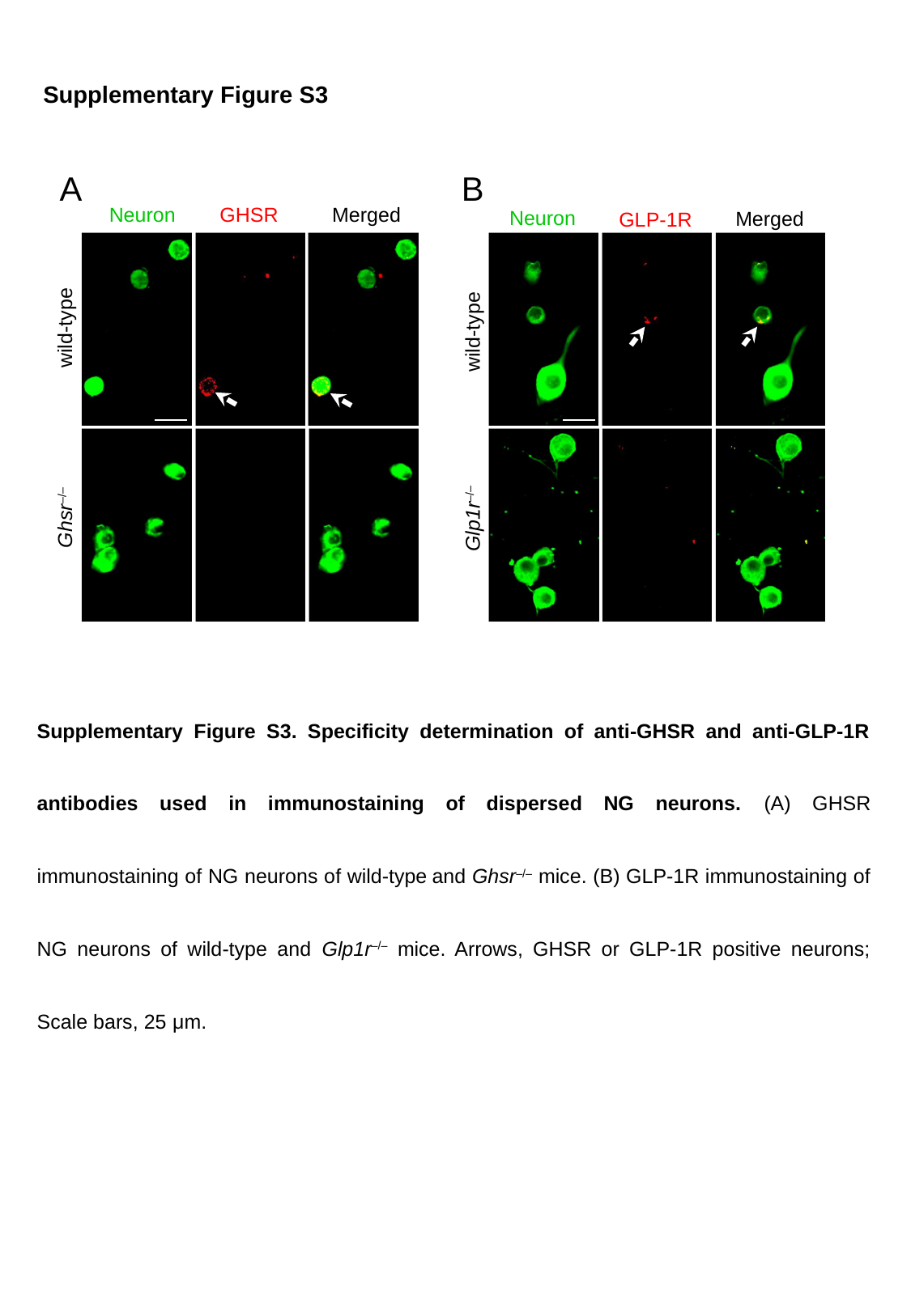

Supplementary Figure S3
A
B
Neuron
GHSR
Merged
wild-type
Ghsr‒/‒
Neuron
Merged
GLP-1R
wild-type
Glp1r‒/‒
Supplementary Figure S3. Specificity determination of anti-GHSR and anti-GLP-1R antibodies used in immunostaining of dispersed NG neurons. (A) GHSR immunostaining of NG neurons of wild-type and Ghsr‒/‒ mice. (B) GLP-1R immunostaining of NG neurons of wild-type and Glp1r‒/‒ mice. Arrows, GHSR or GLP-1R positive neurons; Scale bars, 25 μm.

## Slide 5
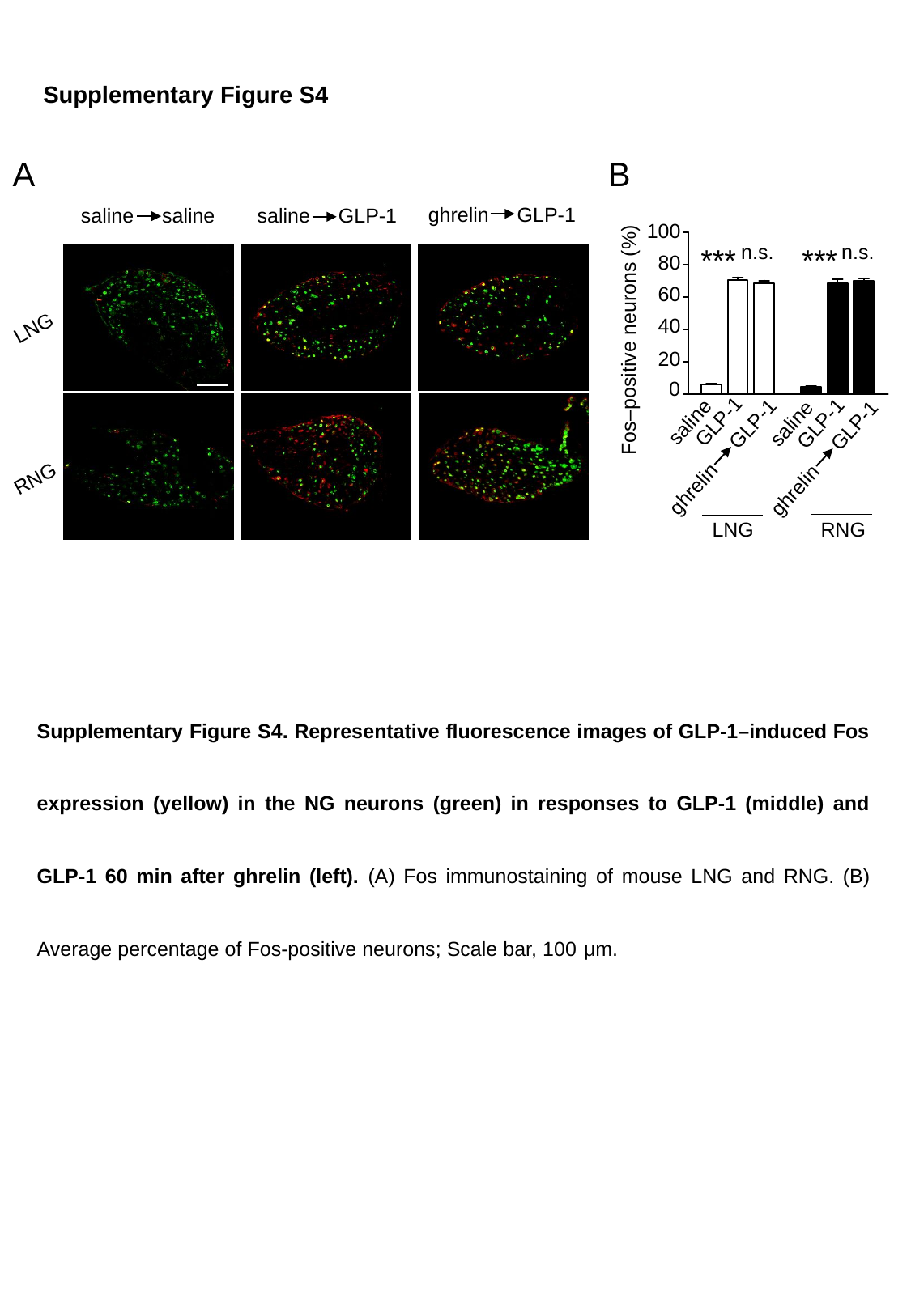

Supplementary Figure S4
A
B
ghrelin GLP-1
saline GLP-1
saline saline
LNG
RNG
100
n.s.
n.s.
***
***
80
60
40
Fos–positive neurons (%)
20
0
saline
GLP-1
saline
GLP-1
ghrelin GLP-1
ghrelin GLP-1
LNG
RNG
Supplementary Figure S4. Representative fluorescence images of GLP-1–induced Fos expression (yellow) in the NG neurons (green) in responses to GLP-1 (middle) and GLP-1 60 min after ghrelin (left). (A) Fos immunostaining of mouse LNG and RNG. (B) Average percentage of Fos-positive neurons; Scale bar, 100 μm.

## Slide 6
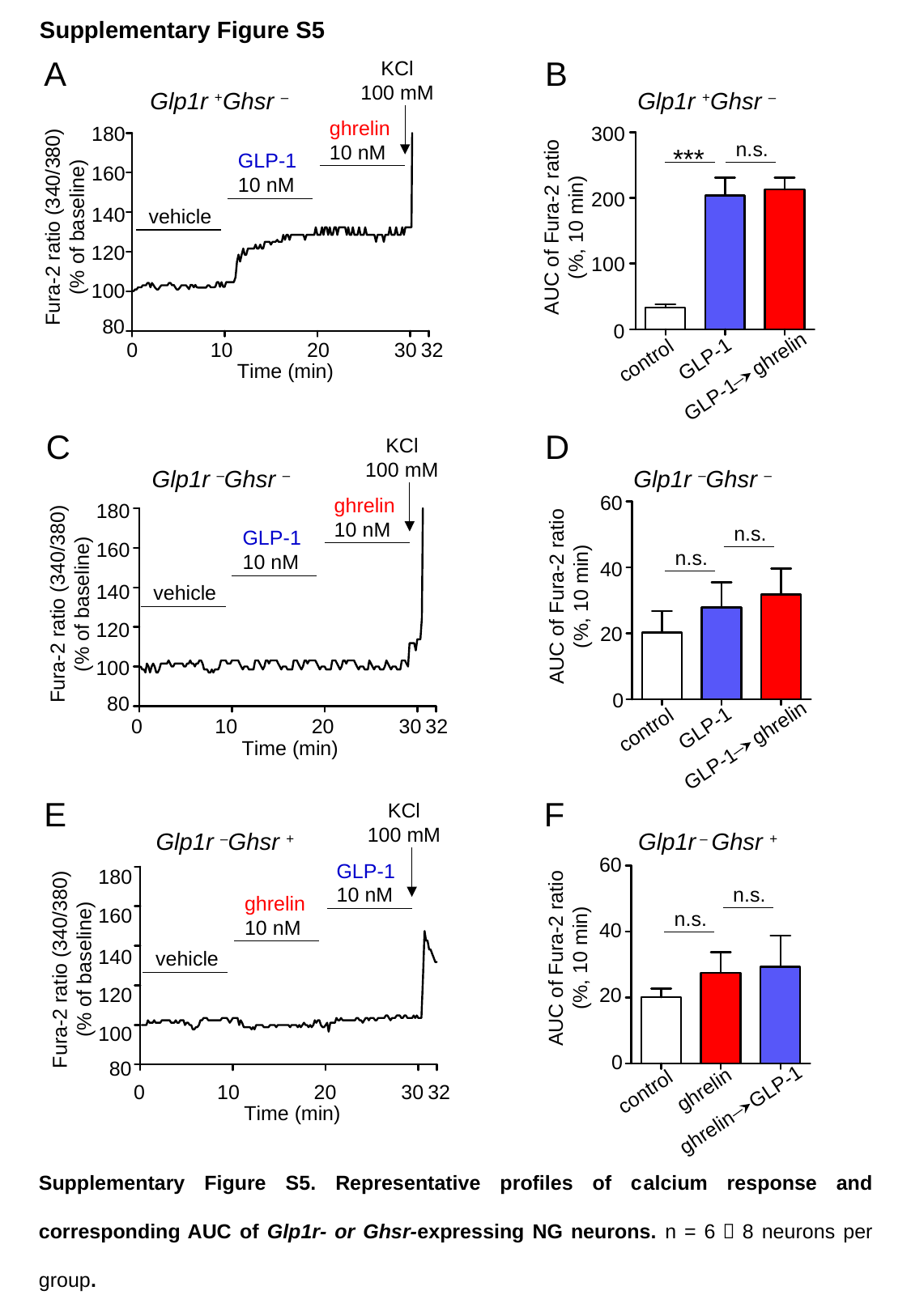

Supplementary Figure S5
A
B
KCl
100 mM
Glp1r +Ghsr ‒
ghrelin
10 nM
180
GLP-1
10 nM
160
140
Fura-2 ratio (340/380)
(% of baseline)
vehicle
120
100
80
0
10
20
30
32
Time (min)
Glp1r +Ghsr ‒
300
n.s.
***
200
AUC of Fura-2 ratio
(%, 10 min)
100
0
GLP-1
control
GLP-1 ghrelin
C
D
KCl
100 mM
ghrelin
10 nM
180
GLP-1
10 nM
160
140
Fura-2 ratio (340/380)
(% of baseline)
vehicle
120
100
80
0
10
20
30
32
Time (min)
Glp1r ‒Ghsr ‒
Glp1r ‒Ghsr ‒
60
n.s.
n.s.
40
AUC of Fura-2 ratio
(%, 10 min)
20
0
GLP-1
control
GLP-1 ghrelin
E
F
KCl
100 mM
GLP-1
10 nM
180
ghrelin
10 nM
160
140
Fura-2 ratio (340/380)
(% of baseline)
vehicle
120
100
80
0
10
20
30
32
Time (min)
Glp1r ‒Ghsr +
Glp1r ‒ Ghsr +
60
n.s.
n.s.
40
AUC of Fura-2 ratio
(%, 10 min)
20
0
ghrelin
control
ghrelin GLP-1
Supplementary Figure S5. Representative profiles of calcium response and corresponding AUC of Glp1r- or Ghsr-expressing NG neurons. n = 6－8 neurons per group.

## Slide 7
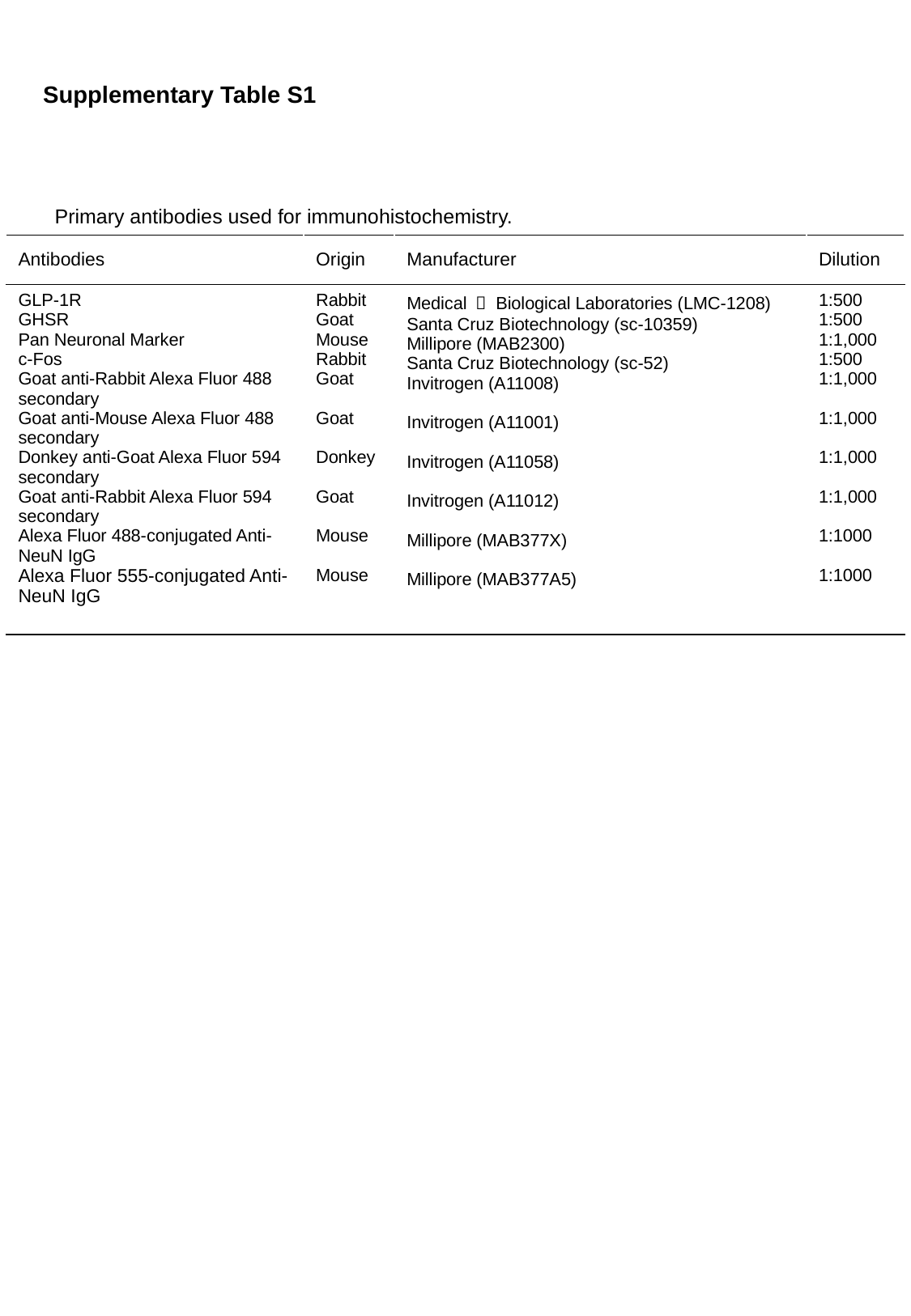

Supplementary Table S1
Primary antibodies used for immunohistochemistry.
| Antibodies | Origin | Manufacturer | Dilution |
| --- | --- | --- | --- |
| GLP-1R GHSR Pan Neuronal Marker c-Fos Goat anti-Rabbit Alexa Fluor 488 secondary Goat anti-Mouse Alexa Fluor 488 secondary Donkey anti-Goat Alexa Fluor 594 secondary Goat anti-Rabbit Alexa Fluor 594 secondary Alexa Fluor 488-conjugated Anti-NeuN IgG Alexa Fluor 555-conjugated Anti-NeuN IgG | Rabbit Goat Mouse Rabbit Goat Goat Donkey Goat Mouse Mouse | Medical ＆ Biological Laboratories (LMC-1208) Santa Cruz Biotechnology (sc-10359) Millipore (MAB2300) Santa Cruz Biotechnology (sc-52) Invitrogen (A11008) Invitrogen (A11001) Invitrogen (A11058) Invitrogen (A11012) Millipore (MAB377X) Millipore (MAB377A5) | 1:500 1:500 1:1,000 1:500 1:1,000 1:1,000 1:1,000 1:1,000 1:1000 1:1000 |

## Slide 8
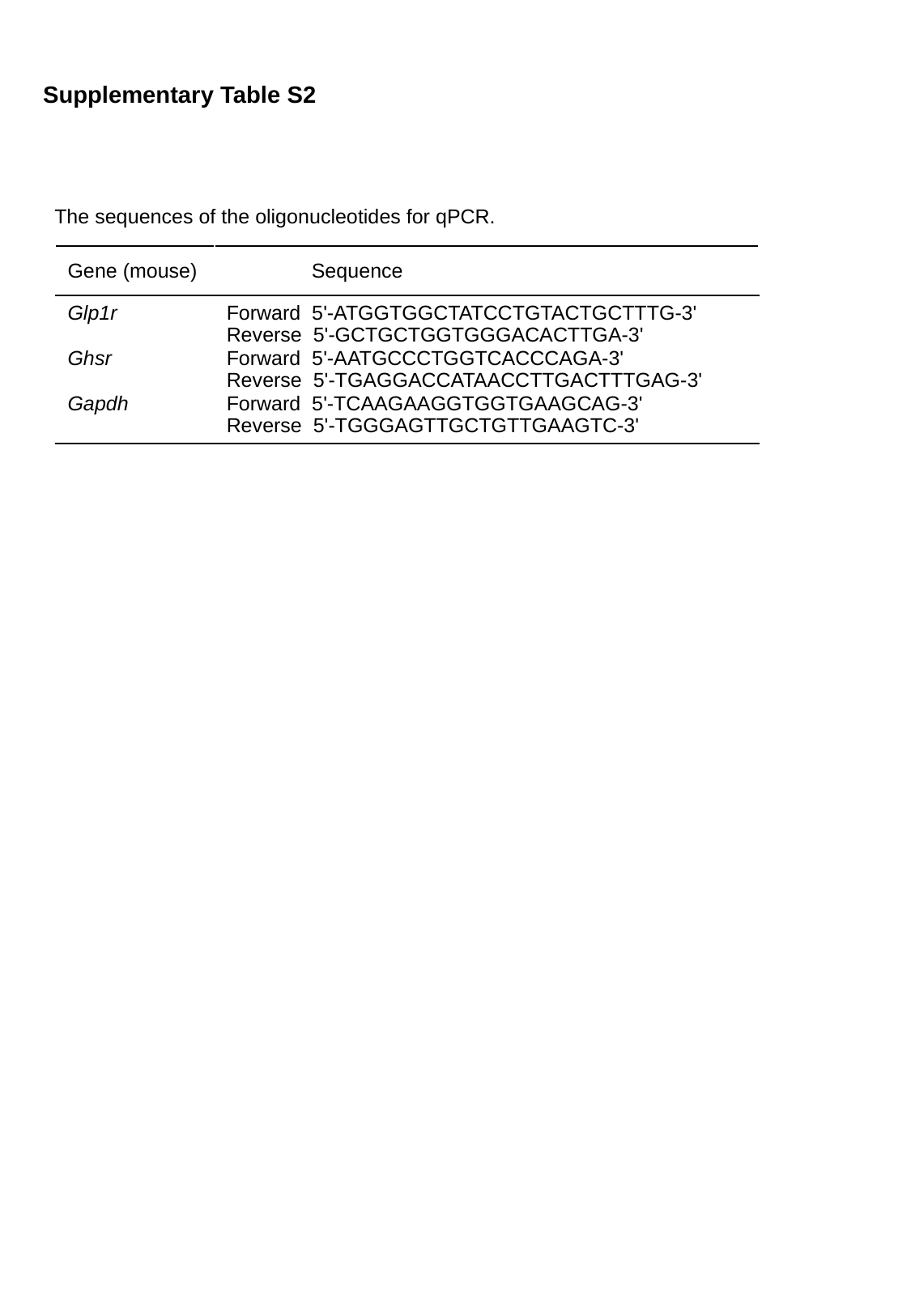

Supplementary Table S2
The sequences of the oligonucleotides for qPCR.
| Gene (mouse) | Sequence |
| --- | --- |
| Glp1r Ghsr Gapdh | Forward 5'-ATGGTGGCTATCCTGTACTGCTTTG-3' Reverse 5'-GCTGCTGGTGGGACACTTGA-3' Forward 5'-AATGCCCTGGTCACCCAGA-3' Reverse 5'-TGAGGACCATAACCTTGACTTTGAG-3' Forward 5'-TCAAGAAGGTGGTGAAGCAG-3' Reverse 5'-TGGGAGTTGCTGTTGAAGTC-3' |
